# Whale Optimization Algorithm for Multiconstraint Second-Order Stochastic Dominance Portfolio Optimization

**DOI:** 10.1155/2020/8834162

**Published:** 2020-08-28

**Authors:** Q. H. Zhai, T. Ye, M. X. Huang, S. L. Feng, H. Li

**Affiliations:** ^1^School of Sciences, Hainan University, No. 58 Renmin Avenue, Haikou 570228, China; ^2^College of Management and Economy, Tianjin University, 92 Weijin Road Nankai District, Tianjin 300072, China; ^3^State Key Laboratory of Marine Resource Utilization in the South China Sea, Hainan University, No. 58 Renmin Avenue, Haikou 570228, China; ^4^School of Information and Communication Engineering, Hainan University, No. 58 Renmin Avenue, Haikou 570228, China

## Abstract

In the field of asset allocation, how to balance the returns of an investment portfolio and its fluctuations is the core issue. Capital asset pricing model, arbitrage pricing theory, and Fama–French three-factor model were used to quantify the price of individual stocks and portfolios. Based on the second-order stochastic dominance rule, the higher moments of return series, the Shannon entropy, and some other actual investment constraints, we construct a multiconstraint portfolio optimization model, aiming at comprehensively weighting the returns and risk of portfolios rather than blindly maximizing its returns. Furthermore, the whale optimization algorithm based on FTSE100 index data is used to optimize the above multiconstraint portfolio optimization model, which significantly improves the rate of return of the simple diversified buy-and-hold strategy or the FTSE100 index. Furthermore, extensive experiments validate the superiority of the whale optimization algorithm over the other four swarm intelligence optimization algorithms (gray wolf optimizer, fruit fly optimization algorithm, particle swarm optimization, and firefly algorithm) through various indicators of the results, especially under harsh constraints.

## 1. Introduction

In the field of finance, the portfolio optimization problem has drawn a lot of attention since the mean-variance (M-V) model was proposed by Markowitz [1]. Based on the probability theory, the M-V model takes the first two moments of the return rate distribution, including expected returns and variance, into account. However, subsequent research studies find that the M-V model is not suitable for the practical financial environment. For example, Chunhachinda et al. [2] pointed out that the returns to the major international stock market are not normally distributed, while the M-V model assumes that the expected returns of the portfolio have a symmetric normal distribution. Besides, the M-V model takes the variance as a risk measure, which counts both upward and downward deviations, which is contrary to the definition of the investment risk [3]. Hence, Markowitz [4] replaced the risk measure with the semivariance, which is more suitable in the case of asymmetric distribution. In addition, there are many other alternative risk measures, such as the mean absolute deviation (MAD), value at risk (VaR), and conditional value at risk (CVaR).

Other than the traditional M-V model, the portfolio optimization model based on the stochastic dominance (SD) relation is highly applied. To be specific, the relationship between the first-order stochastic dominance (FSD) and utility theory was first discussed by Quirk and Saposnik [5]. Hadar and Russell [6] extended the above relationship to the second-order stochastic dominance (SSD). Thereinto, different-order SD relations relate to different kinds of utility functions. In the meantime, SD relation is of interest because it takes the risk appetite into consideration and is more suitable to investors in realistic financial environments. Specifically speaking, FSD reflects the behavior of rational investors, while SSD reflects the behavior of rational risk-averse investors. Dentcheva and Ruszczynski [7] first proposed the portfolio optimization model with SD constraints. Then, Roman and Mitra [8] pointed out that the portfolio optimization model with the SSD rule improves the performance of the traditional M-V model. Besides, Leshno and Levy [9] established an almost stochastic dominance (ASD) relation, which reveals the preference for most investors rather than all of them. Moreover, Fabian et al. [10] proposed the SSD portfolio optimization model, which is called TSSD, using tail risk measures at different confidence levels. Recently, Javanmardi and Lawryshyn [11] proposed the SSD-DP model, which does not require a benchmark portfolio.

However, only considering the SSD constraint is not enough to get an effective portfolio in the complex investment environment. Therefore, additional real-life constraints, such as transaction costs, higher moments, diversification, and boundary constraints, should be taken into consideration in portfolio optimization. In any financial market, investors are obliged to pay transaction costs when buying or selling the securities. As one of the key factors affecting the net return, transaction costs are of great importance to investors, and ignoring them will result in an ineffective portfolio [12, 13]. Besides, higher moments of return distribution, including skewness and kurtosis, have drawn investors' attention, which takes the extreme value into account rather than focusing solely on the average [14]. Leung et al. [15] pointed out that neglecting skewness will lead to an inefficient portfolio. Yu and Lee [16] showed that the model with higher moments performs better. As for diversification, it is a strategy that integrates a wide variety of investments into the portfolio. Models considering diversity are propitious to eliminate the unsystematic risk in the portfolio and make asset allocation more feasible [17, 18]. Normally, Shannon's entropy and Yager's entropy are used to measure the diversity of a portfolio. Furthermore, given that small proportions held in the portfolio have little impact on its performance and high proportions go against the flexibility, the boundary constraint, also known as the buy-in threshold constraint, is included in the portfolio optimization model to limit the upper and lower bounds of the investment ratio in individual assets [19, 20].

After introducing several constraints into the model, the portfolio optimization problem converts to an NP-complete problem, which limits the computational efficiency as the problem size increases. Besides, SSD constraints require the comparison of any two investable assets. As the number of investable assets increases, the computational requirements become more demanding. Therefore, heuristic algorithms are widely used to optimize the multiconstraint portfolio optimization problem, such as the particle swarm optimization (PSO) [21], firefly algorithm (FA) [22], biogeography-based optimization (BBO), and artificial bee colony (ABC) [23]. Recently, Babazadeh and Esfahanipour [24] developed a nondominated sorting genetic algorithm and applied it to a multiperiod mean-var portfolio optimization model under the cardinality, budget, floor, and ceiling constraints. Chen et al. [25] combined the FA and the genetic algorithm (GA) and then applied it to a mean-variance-skewness portfolio selection model under the transaction costs, bounds on holdings, cardinality, and transaction lots constraints.

Whale optimization algorithm (WOA) is a metaheuristic optimization algorithm introduced by Mirjalili and Lewis [26], which is inspired by the bubble-net hunting strategy of humpback whales. Besides, WOA was tested for 29 mathematical optimization problems and 6 structural design problems, and the results prove that WOA is very competitive with the existing metaheuristic algorithms as well as conventional methods. The most important is that WOA mainly mimics the hunting behavior of humpback whales in searching for and attacking preys called the bubble-net feeding behavior [27], which improved the performance of candidate solutions in each step. Because of its characteristics of simple theory, easier operation, less parameter setting, and no special requirements for optimized function, WOA is widely used in image segmentation [28], parameter estimation [29], sizing optimization [30], and global optimization [31]. Recently, Abdel-Basset et al. [32] integrated WOA with a local search strategy for solving the problem of permutation flow shop scheduling. Reddy et al. [33] applied WOA to profit-based unit commitment problems in competitive electricity markets with good performance.

However, in these papers, WOA has still not been applied to solving the multiconstraint SSD portfolio optimization model. The motivation for proposing WOA for the multiconstraint SSD portfolio optimization model in this research is twofold. First, based on the above literature review, there have been several successful applications based on WOA. Second, in the preliminary research work, we have achieved decent performance on the SSD portfolio optimization model by GWO. In this paper, a new improved multiconstraint SSD portfolio optimization model is proposed and optimized by WOA.

For the multiconstraint SSD portfolio optimization model, the area of the feasible region is very small, which makes it difficult to produce feasible solutions. At the same time, considering harsh constraints makes the traditional optimization methods tend to converge to the local optimum, which results in the loss of population diversity and poor optimization ability. Compared with the traditional evolutionary algorithm, such as GA, on the one hand, WOA has a stronger ability to jump out of the local optimum and explore the globally optimal solution; on the other hand, the bubble-net attack process ensures that WOA has higher accuracy than other swarm intelligence algorithms, such as the gray wolf optimizer (GWO) and the fruit fly optimization algorithm (FOA). Therefore, WOA is used to optimize the above multiconstraint SSD portfolio optimization model. Furthermore, we perform numerical experiments based on FTSE100 index data and compare the performance of the optimal portfolio obtained by WOA with that obtained by GWO, FOA, PSO, and FA.

The novel contributions of this paper are as follows.

Firstly, the multiconstraint SSD portfolio optimization model is proposed. We incorporate several realistic constraints into the SSD portfolio optimization model, including the transaction cost, skewness, kurtosis, diversification, and boundary constraints. Secondly, WOA is successfully used to optimize the above multiconstraint SSD portfolio optimization model. Lastly, an optimal portfolio strategy obtained by WOA is found to be greater than that obtained by other four different algorithms. The rest of this paper is organized as follows. In Section 2, we discuss the portfolio optimization model with SSD and other real-life constraints. In Section 3, we discuss the WOA for solving the proposed model. In Section 4, we present a numerical experiment and analyse the performance of WOA. Finally, a brief conclusion is illustrated in Section 5.

## 2. The Multiconstraint Portfolio Optimization Model

In this section, a detailed discussion of the SSD portfolio optimization model under several realistic or higher moment constraints is presented. Specifically speaking, it is well known that only considering returns and risk is not enough to get an effective portfolio that meets the complicated investment environment. Besides, in the real-world financial market, the return distribution of the portfolio is not a normal distribution, which means that higher moments of return distribution should not be neglected. Therefore, several realistic or higher moment constraints, including the transaction cost, skewness, kurtosis, diversification, and boundary constraints, are included in the framework of SSD portfolio optimization.

### 2.1. The Returns, Net Return, and Excess Mean Return

As one of the most basic elements of portfolio optimization problems, returns play an important part in indicating the performance of a portfolio. In the financial market, the returns of investment can be defined as the ratio of the net gain or loss to the initial cost of the investment, separately known as positive returns and negative returns, which can be formulated as follows:

(1) $$\begin{array}{c} r_{i,t}=\frac{p_{i,t}-p_{i,t-1}+d_{i,t}}{p_{i,t-1}}, \end{array}$$

where *p*_*i*,*t*_ and *p*_*i*,*t*−1_ are the price of asset *i* at period *t* and *t* − 1, respectively, and *d*_*i*,*t*_ is the dividend of asset *i* and period *t*. Let *n* denote the number of assets, which are available for investment at the beginning of a fixed period and *x*=(*x*_1_, *x*_2_,…, *x*_*n*_) denote the fraction of the initial capital invested in *x*_*i*_. Then, we use *X* ∈ *R*^*n*^ to denote a set of feasible portfolios, which is a bounded convex polyhedron clearly. Besides, let *R*_*i*_(*ξ*) denote the returns of asset *i* under discrete distribution, where a random vector is on the probability space (Ω, *F*, *P*). Assuming that the capital available for investment is fixed and *E*[|*R*_*j*_|] < *∞*, the returns of portfolio *x* can be expressed as follows [3]:

(2) $$\begin{array}{c} g\left(x,\xi \right)=\sum_{i=1}^{n}R_{i}\left(\xi \right)x_{i}. \end{array}$$

However, we always need to pay fees when trading assets, such as brokerage fees, bid-ask spreads, taxes, and fund load, which are known as transaction costs. Arnott and Wanger [12] pointed out that ignoring the transaction costs would lead to an ineffective portfolio. In this paper, we employ a V-shaped transaction cost function to express the transaction costs between the new portfolio *x*=(*x*_1_, *x*_2_,…, *x*_*n*_) and the existing portfolio *x*_0_=(*x*_1_^0^, *x*_2_^0^,…*x*_*n*_^0^). Let *c*(*x*) denote the total transaction costs of *n* assets, which can be formulated as follows:

(3) $$\begin{array}{c} c\left(x\right)=\sum_{i=1}^{n}c\left(i\right)\left|x_{i}-x_{i}^{0}\right|, \end{array}$$

where *c*(*i*) is the unit transaction cost of asset *i*. It is set to 0.9%, among which the commission is 0.4% and the stamp duty is 0.5%. Thus, the net return of portfolio *x* can be displayed as follows [34]:

(4) $$\begin{array}{c} f\left(x,\xi \right)=\sum_{i=1}^{n}R_{i}\left(\xi \right)x_{i}-\sum_{i=1}^{n}c\left(i\right)\left|x_{i}-x_{i}^{0}\right|. \end{array}$$

Furthermore, the excess mean return (EMR) is always used to describe performance over the existing portfolio, which is an average of the difference between the returns of the new portfolio *x*=(*x*_1_, *x*_2_,…, *x*_*n*_) and the existing portfolio *x*_0_=(*x*_1_^0^, *x*_2_^0^,…*x*_*n*_^0^), and can be formulated as follows:

(5) $$\begin{array}{c} \text{EMR}\left(x\right)=\frac{1}{T}\sum_{j=1}^{T}\left(R_{j}\left(x\right)-R_{j}\left(x_{0}\right)\right), \end{array}$$

where *R*_*j*_(*x*) and *R*_*j*_(*x*_0_) denote the returns of the portfolio *x* and *x*_0_ in the period *j*. In a word, all of the above three indicators can be used to evaluate the performance of a new portfolio. Specifically speaking, the returns of portfolio are used to calculate constraints, such as skewness. The net return of the portfolio is used as an objective function, and the EMR is only used as an evaluation indicator.

Fama and French [35] put forward a three-factor pricing model and recently put forward a five-factor pricing model [36]. It builds its portfolio at the end of June and holds it for a year, based on financial data from the previous year. In order to simplify the rebalancing problem, this paper only considers the construction of a single-period portfolio and assumes that the transaction cost required to construct each portfolio is fixed. Therefore, the returns of portfolio are used as the objective function of the optimization model in this paper.

### 2.2. Risk Measures and Stochastic Dominance Constraint

As another basic element of portfolio optimization problems, risk presents the uncertainty of returns. The risk measures have many forms, including variance, semivariance (SV), MAD, VaR, and CVaR. VaR is one of the most well-known downside risk measures, which measures the worst returns a portfolio may potentially suffer. With a fixed confidence level *α*, VaR_*α*_ is defined as the *α*-quantile of the cumulative distribution function, which can be defined as follows:

(6) $$\begin{array}{c} {\text{VaR}}_{\alpha }\left(Y\right)=\inf\left\{\left.u\right|\text{prob}\left\{Y\leq u\right\}\geq \alpha \right\}, \end{array}$$

where *Y* represents the returns of the portfolio in each period. Meanwhile, as a coherent risk measure, CVaR measures the conditional expectation of losses beyond VaR, which can be displayed as follows:

(7) $$\begin{array}{c} {\text{CVaR}}_{\alpha }\left(Y\right)=E\left(\left.Y\right|Y\leq {\text{VaR}}_{\alpha }\left(Y\right)\right). \end{array}$$

Unlike the above risk measures, the SD constraint is not an indicator, which establishes relative risk advantages by comparison between the *k*-order distribution function of any two portfolios. Assuming that the utility function of all investors is monotonically increasing, the portfolio *Y* stochastically dominates the portfolio *Y* in the first order when all the investors prefer portfolio *X* to portfolio *X* or that there is no difference between a part of them [37]. Let *x* and *x*^0^ be the decision vectors and *ξ* be a random variable; then *g*(*x*, *ξ*) is preferred to *g*(*x*^0^, *ξ*) weakly in *E*[(*η* − *X*)_+_] ≤ *E*[(*η* − *X*^0^)_+_], ∀*η* ∈ *ℝ*, first-order stochastic dominance, denoted by $g\left(x,\xi \right){\underline{≻}}_{\left(1\right)}g\left(x^{0},\xi \right)$, if and only if

(8) $$\begin{array}{c} F\left(g\left(x,\xi \right);\eta \right)\leq F\left(g\left(x^{0},\xi \right);\eta \right),\;\forall \eta \in \mathbb{R}, \end{array}$$

where *g*(*x*, *ξ*) is the returns of the portfolio *x* ∈ *R*_*n*_, which is a concave continuous function both in *x* and *ξ*, and *F*(*g*(*x*, *ξ*); *η*) is the cumulative distribution function of *g*(*x*, *ξ*). Similarly, *g*(*x*, *ξ*) is preferred to *g*(*x*^0^, *ξ*) weakly in the SSD, denoted by $g\left(x,\xi \right){\underline{≻}}_{\left(2\right)}g\left(x^{0},\xi \right)$, if and only if

(9) $$\begin{array}{c} \int_{-\infty }^{\eta }F\left(g\left(x,\xi \right);\alpha \right)\text{d}\alpha \leq \int_{-\infty }^{\eta }F\left(g\left(x^{0},\xi \right);\alpha \right)\text{d}\alpha ,\;\forall \eta \in \mathbb{R}. \end{array}$$

Therefore, the strict dominance relation *succ*_*k*_ is defined as follows:

(10) $$\begin{array}{c} X{≻}_{\left(k\right)}X^{0}⟺X{≽}_{\left(k\right)}X^{0},\\ X{\underline{⋡}}_{\left(k\right)}X^{0}. \end{array}$$

There are several equivalent characterizations of the SSD constraint. Hadar and Russell [6] pointed out that for any nondecreasing and concave utility function *u* ∈ *U*={*U* : *U*′ ≥ 0, *U*^″^ ≤ 0}, *X*≻_(2)_*X*^0^ if and only if

(11) $$\begin{array}{c} E\left[u\left(X\right)\right]\geq E\left[u\left(X^{0}\right)\right], \end{array}$$

where *X* and *X*^0^ are the two random variables, which generally represent the returns of portfolios *x* and *x*^0^, and *E*(·) is the expected value with respect to the probability distribution of *ξ* [10]. Ogryczak and Ruszczyński [38] pointed out that *X*≻_(2)_*X*^0^ if and only if

(12) $$\begin{array}{c} E\left[{\left(\eta -X\right)}_{+}\right]\geq E\left[{\left(\eta -X^{0}\right)}_{+}\right],\;\forall \eta \in \mathbb{R}, \end{array}$$

where *E*[(*η* − *X*)_+_]=*E*(max{*η* − *X*, 0}). Ogryczak and Ruszczyński pointed [39] out that *X*≻_(2)_*X*^0^ is equivalent to the continuum of CVaR constraints for all confidence levels *α* ∈ [0,1]:

(13) $$\begin{array}{c} {\text{CVaR}}_{\alpha }\left(R_{x}\right)\geq {\text{CVaR}}_{\alpha }\left(R_{y}\right),\;\forall \alpha \in \left(0,1\right]. \end{array}$$

In this paper, we use the last equivalent SSD relation, VaR, and CVaR as evaluation indicators.

### 2.3. Higher Moment Constraints

The traditional M-V model formulated by Markowitz [1] only takes the first two moments of return distribution into account for portfolio optimization. Arditti and Levy [40] and Rubinstein [41] have argued that the higher moments of return distribution should not be neglected because the distribution of returns in the financial market is not a normal distribution. From the theoretical and empirical point of view, Arditti [42, 43] proved that investors demand higher (lower) returns for investments with negative (positive) skewness of income distribution. Further, Scott and Horvath [44] extended this analysis to the higher-order moments of the return distribution and proved that the positive values of even (odd) order moments bring a positive (negative) risk premium, and vice versa. Referring to the recent studies by Chen and Yue et al. [25, 45], the skewness and kurtosis constraints are incorporated into the optimization model in this paper.

Let *ζ* be an uncertain variable with a finite expected value *e*, and the skewness and kurtosis of *ζ* are, respectively, defined by

(14) $$\begin{array}{c} S\left[\zeta \right]=E\left[{\left(\zeta -e\right)}^{3}\right],\\ K\left[\zeta \right]=E\left[{\left(\zeta -e\right)}^{4}\right]. \end{array}$$

### 2.4. Diversification Constraint

In the financial market, portfolio diversification is the process of allocating the capital in a way that reduces the exposure to any one particular asset or risk, which implies that the idiosyncratic risk of the portfolio can be reduced as the assets included in the investment increase. Considering the low diversity of the portfolio which may lead to losses, we include the diversification constraint into the portfolio optimization model. SE and Yager's entropy are widely used to measure the diversity of a portfolio [17, 46]. Let *x*(*i*) denote the weight of assets *i*, and SE can be formulated as follows:

(15) $$\begin{array}{c} \text{SE}\left(x\right)=-\sum_{i=1}^{n}x_{i}\ln x_{i}, \end{array}$$

and Yager's entropy can be calculated as follows:

(16) $$\begin{array}{c} \text{YE}\left(x\right)=-{\left({\sum_{i=1}^{n}\left|x_{i}-\frac{1}{n}\right|}^{z}\right)}^{\left(1/z\right)}, \end{array}$$

where *z* ≥ 1 is a constant, and in this paper, it is set to 2. Obviously, the higher the value of SE or Yager's entropy, the better the diversity of the portfolio. In particular, both SE and Yager's entropy get the maximum value when *x*(*i*)=(1/*n*).

### 2.5. Boundary Constraint

The boundary constraint, also known as the buy-in threshold constraint, means that each asset should be invested in a specific range, while the lower bounds are used to reduce the brokerage costs and monitoring costs and upper bounds are used to increase the flexibility [19]. Let *ε*_*i*_ denote the lower bounds and *δ*_*i*_ denote the upper bounds; then the boundary constraint of the portfolio *x* can be described as follows:

(17) $$\begin{array}{c} \varepsilon_{i}z_{i}\leq x_{i}\leq \delta_{i}z_{i}, \end{array}$$

where *x*_*i*_ is the weight of assets *i* and *z*_*i*_ ∈ {0,1}; if *x*_*i*_ > 0, *z*_*i*_=1; otherwise, *z*_*i*_=0.

### 2.6. Budget Constraint

Budget constraint means that the amount of capital to be invested is fixed and all capital should be invested, which can be expressed as follows:

(18) $$\begin{array}{c} \sum_{i=1}^{n}x_{i}=1. \end{array}$$

### 2.7. No Short Selling Constraint

The short selling refers to the sale of securities borrowed by the seller, which is an act of speculation with high risk in the financial market. Therefore, the short selling is not considered in this paper, and the no short selling constraint is represented by

(19) $$\begin{array}{c} x_{i}\geq 0. \end{array}$$

### 2.8. Multiconstraint Second-Order Stochastic Dominance Portfolio Optimization Model

Given all that, the multiconstraint SSD portfolio optimization model is provided below:

(20) $$\begin{array}{c} \begin{array}{cc} \max & f\left(x,\xi \right):=\overset{n}{\underset{i=1}{\sum }}R_{i}\left(\xi \right)x_{i}-\sum_{i=1}^{n}c\left(i\right)\left|x_{i}-x_{i}^{0}\right|,\\ \text{s.t.} & \int_{-\infty }^{\eta }F\left(g\left(x,\xi \right);\alpha \right)d\alpha \leq \int_{-\infty }^{\eta }F\left(g\left(x,\xi^{0}\right);\alpha \right)d\alpha ,\;\forall \eta \in \mathbb{R},\\ & S\left(x\right)>S\left(x^{0}\right),\\ & K\left(x\right)<K\left(x^{0}\right),\\ & \text{SE}\left(x\right)>\text{SE}\left(x^{0}\right),\\ & \varepsilon_{i}z_{i}\leq x_{i}\leq \delta_{i}z_{i},\;z_{i}\in \left\{0,1\right\},\\ & \overset{n}{\underset{i=1}{\sum }}x_{i}=1,\;x_{i}\geq 0,i=1,2,\ldots ,n. \end{array} \end{array}$$

## 3. The Whale Optimization Algorithm (WOA) for Solving the Proposed Model

### 3.1. Background

**Figure 1:**
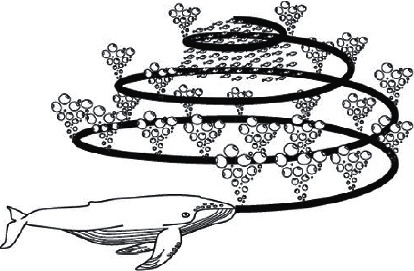
Bubble-net feeding behavior of humpback whales.

WOA is a swarm intelligence optimization algorithm proposed by Australian scholars Mirjalili and Lewis [26] in 2016. The algorithm simulates the process of humpback whales searching and capturing food by establishing mathematical models. WOA was inspired by the bubble-net attack strategy of humpback whales. Whales surround their prey by spiraling up and bubbling up as they dive about 12 meters below the surface. WOA has the advantages of a simple principle, few parameters, and strong searching ability. Since its inception, it has been widely used in engineering optimization, parameter extraction, feature selection, and other such aspects.  Figure 1 shows the bubble-net feeding behavior of humpback whales.

### 3.2. Mathematical Model

In WOA, the position of the *i*th whale (search agent) is described as *P*_*i*_=[*X*_*i*,1_, *X*_*i*,2_,…, *X*_*i*,*j*_], where *i* is required to be given in advance, and the size of *j* is equal to dim (the dimension of the problem). The steps of the WOA are as follows.

First, initial search agents with positions [0,0,…, 0] are generated. Then, the position and the score of the optimal search agent with position *P*_*∗*_=[0,0,…, 0] are initialized. For solving the minimum problem, the bestscore (the initial score of the optimal search agent) is set to be +*∞* instead of −*∞*.

The algorithm loop is entered. For each search agent, each variable *X*_*i*,*j*_(*j*=1,2,…, dim) is checked whether or not it is across the border. For a variable that is out of bounds, its value is returned to the boundary.

Real numbers $\overset{⟶}{r_{1}}$ and $\overset{⟶}{r_{2}}$ are randomly generated and *p* ∈ [0,1] and are used to calculate $\overset{⟶}{A}$ and $\overset{⟶}{C}$. The calculation formulas of $\overset{⟶}{A}$ and $\overset{⟶}{C}$ are as follows:

(21) $$\begin{array}{c} \overset{⟶}{A}=2\cdot \overset{⟶}{a}\cdot \overset{⟶}{r_{1}}-\overset{⟶}{a}, \end{array}$$

(22) $$\begin{array}{c} \overset{⟶}{C}=2\cdot \overset{⟶}{r_{2}}, \end{array}$$

where $\overset{⟶}{a}$ is a real number that goes linearly from 2 to 0 in the iteration.

#### 3.2.1. Random Prey

If *p* < 0.5 and $\left|\overset{⟶}{A}\right|>1$, the stage of random search for the prey is entered. In this process, the search agent is forced to move away from its current location and wander randomly through the space in search of the prey. The mathematical model and position transformation formula are as follows:

(23) $$\begin{array}{c} \overset{⟶}{D}=\left|\overset{⟶}{C}\times \overset{⟶}{P_{\text{rand}}}-\overset{⟶}{P_{i}^{t}}\right|, \end{array}$$

(24) $$\begin{array}{c} \overset{⟶}{P_{i}^{t+1}}=\overset{⟶}{P_{\text{rand}}}-\overset{⟶}{A}\times \overset{⟶}{D}, \end{array}$$

where $\overset{⟶}{P_{\text{rand}}}$ is a randomly generated position vector within the boundary range, $\overset{⟶}{P_{i}^{t}}$ is the generation *t* of the *i*th search agent's position vector, and $\overset{⟶}{P_{i}^{t+1}}$ is the *i*th search agent's position vector generation *t*+1.

#### 3.2.2. Encircling Prey

If *p* < 0.5 and $\left|\overset{⟶}{A}\right|\leq 1$, the stage of surrounding the prey is entered. In this process, the location of the prey is identified, and the prey is surrounded. The search agent moves closer to the location of the optimal search agent. The mathematical expression of its position update is

(25) $$\begin{array}{c} \overset{⟶}{D}=\left|\overset{⟶}{C}\times \overset{⟶}{P_{*}^{t}}-\overset{⟶}{P_{i}^{t}}\right|,\\ \overset{⟶}{P_{i}^{t+1}}=\overset{⟶}{P_{i}^{t}}-\overset{⟶}{A}\times \overset{⟶}{D}, \end{array}$$

where $-P\overset{⟶}{P_{*}^{t}}$ is the generation *t* of optimal search agent's position vector.

#### 3.2.3. Bubble-Net Attacking

If *p* ≥ 0.5, the stage of spiral contraction encircling and bubble-net attack is entered. Firstly, the distance between the search agent and the optimal search agent is calculated, and then a spiral mathematical model is established to update the search agent's position. The formula is as follows:

(26) $$\begin{array}{c} \overset{⟶}{P_{i}^{t+1}}=\overset{⟶}{D^{\prime }}\cdot e^{bl}\cdot \cos\left(2\pi l\right)+\overset{⟶}{P_{*}^{t}}, \end{array}$$

where $\overset{⟶}{D^{\prime }}=\left|\overset{⟶}{P_{*}^{t}}-\overset{⟶}{P_{i}^{t+1}}\right|$, *b* is the defined logarithmic helix shape constant, and *t* is a random real number in the range [−1,1].

### 3.3. The WOA for Solving the Proposed Model

**Figure 2:**
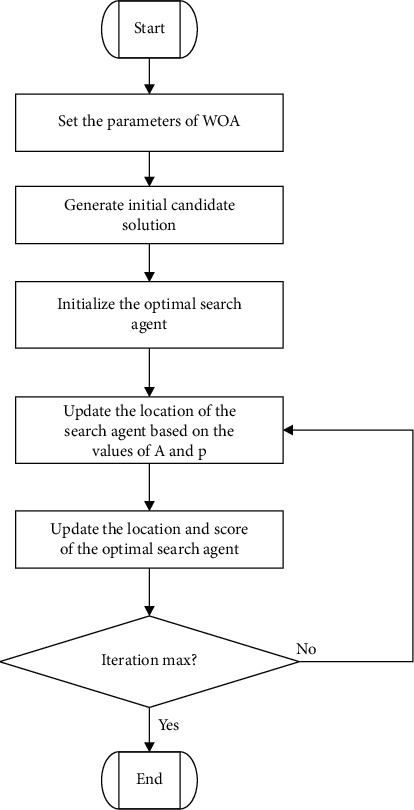
The procedure of the WOA algorithm.

When it comes to WOA for solving this model, we need to add judgment constraints into the WOA iteration process. That is, the search agent that meets the constraint and has better fitness is allowed to be updated to the leading search agent. There are several constraints in the model. In order to ensure the quality of the final solution, we added the initialization coefficient *β*=1.5 in the initialization process, which makes the quality of the initial solution reach our desired effect.  Figure 2 shows the procedure of the WOA algorithm. Meanwhile, the pseudocode of the main procedure of WOA is shown in Algorithm 1, and the pseudocode of WOA for solving the proposed model is shown in Algorithm 2.

## 4. Numerical Experiments

In this section, we carry out several numerical experiments based on FTSE100 index stock historical data. Specifically speaking, the historical return rate of FTSE 100 index assets prior to December 2018 is collected to construct the portfolio strategy. Besides, a series of indicators are introduced to evaluate the performance of portfolios by Goel and Sharma [47], such as EMR, downside deviation (DD), and Sharpe ratio. Moreover, the algorithms are coded in MATLAB 2016a, and all tests are performed on a PC with a Windows 10 operating system and 8 GB of RAM.

### 4.1. Performance Measures

We mainly evaluate a portfolio from two aspects: absolute indicator and relative indicators, which are described briefly as follows.

As a measure of the downside risk, DD focuses on the returns below the minimum threshold or the minimum acceptable returns. Let *R*_*j*_(*x*) and *R*_*j*_(*x*_0_) denote the returns of portfolios *x* and *x*_0_ in the period *j*; then DD can be formulated as follows:

(27) $$\begin{array}{c} DD\left(x\right)=\sqrt{\frac{1}{T}\sum_{j=1}^{T}\min{\left[R_{j}\left(x\right)-R_{j}\left(x_{0}\right),0\right]}^{2}}, \end{array}$$

where *R*_*j*_(*x*_0_) is the FTSE100 index in this paper, and the lower value of DD means a better performance of portfolio *x*. Besides, DD is also used to calculate the Sortino ratio. Sortino ratio is a variation of the Sharpe ratio, which uses DD rather than the standard deviation as a risk measure. The Sortino ratio and the Sharpe ratio can be calculated as follows:

(28) $$\begin{array}{c} \text{Sortino ratio}\left(x\right)=\left\{\begin{array}{cc} \frac{\text{EMR}\left(x\right)}{DD}, & \text{EMR}>0,\\ 0, & \text{EMR}\leq 0, \end{array}\right.\\ \text{Sharpe ratio}\left(x\right)=\left\{\begin{array}{cc} \frac{R\left(x\right)-R_{f}}{\sigma \left(R\left(x\right)\right)}, & R\left(x\right)-R_{f}\geq 0,\\ 0, & R\left(x\right)-R_{f}<0, \end{array}\right. \end{array}$$

where *R*(*x*) denotes the net return of portfolio *x*, *R*_*f*_ denotes the risk-free rate, and *σ*(·) denotes the standard deviation. Obviously, a higher value of the Sortino ratio or the Sharpe ratio is desirable. Moreover, the STARR ratio (STARR) and the information ratio (IR) are alternatives to the Sharpe ratio. STARR also considers the major drawback of the standard deviation as a risk measure and employs the CVaR for the performance adjustment, which is defined as

(29) $$\begin{array}{c} \text{STAR}R_{\alpha }\left(x\right)=\left\{\begin{array}{cc} \frac{\text{EMR}\left(x\right)}{CVaR_{\alpha }\left(R\left(x\right)-R\left(x_{0}\right)\right)}, & \text{EMR}>0,\\ 0, & \text{EMR}\leq 0, \end{array}\right. \end{array}$$

where *α* is the confidence level. Considering that CVaR is usually negative, therefore, the lower value of STARR is preferable. Furthermore, IR is a measure of the returns on a portfolio beyond a benchmark, and the benchmark is typically an index, such as the FTSE100 index, which is given by

(30) $$\begin{array}{c} \text{IR}\left(x\right)=\left\{\begin{array}{cc} \frac{\text{EMR}\left(x\right)}{\sigma \left(R\left(x\right)-R\left(x_{0}\right)\right)}, & \text{EMR}>0,\\ 0, & \text{EMR}\leq 0. \end{array}\right. \end{array}$$

Similarly, the higher value of IR is desirable. In this paper, the confidence level *α* is set to 5%, the risk-free rate *R*_*f*_ is set to 4%, and the FTSE100 index is used as a benchmark.

### 4.2. Numerical Results

In this section, we report the detailed experimental results. Specifically, the return rate of FTSE100 index stocks during 2018 is collected as historical data. Besides, in order to ensure the diversity of the portfolio, the upper bound *δ* of the portfolio is set to 5%, 7%, or 10%. Above all, we propose the following multiconstraint portfolio optimization model under SSD:

(31) $$\begin{array}{c} \begin{array}{cc} \max & f\left(x,\xi \right):=\overset{n}{\underset{i=1}{\sum }}R_{i}\left(\xi \right)x_{i}-\sum_{i=1}^{n}c\left(i\right)\left|x_{i}-x_{i}^{0}\right|,\\ \text{s.t.} & \int_{-\infty }^{\eta }F\left(g\left(x,\xi \right);\alpha \right)\text{d}\alpha \leq \int_{-\infty }^{\eta }F\left(g\left(x^{0},\xi \right);\alpha \right)\text{d}\alpha ,\;\forall \eta \in R,\\ & S\left(x\right)>S\left(x^{0}\right),\\ & K\left(x\right)<K\left(x^{0}\right),\\ & \text{SE}\left(x\right)>\text{SE}\left(x^{0}\right),\\ & x\in X=\left\{\left.\left(x_{1},\ldots ,x_{n}\right)\right|\overset{n}{\underset{i=1}{\sum }}x_{i}=1,\;0\leq x_{j}\leq \sigma ,\forall j\in 1,\ldots ,n\right\}. \end{array} \end{array}$$

To evaluate the performance of the WOA, we compare it with the standard GWO, FOA, PSO, and FA. In order to make the running time roughly equal, except for FA, the number of initial solutions for the other four algorithms is 3000 (in FA, it is set to 1500). And, the initialization coefficient *β*=1.5. In WOA, GWO, and PSO, the number of iterations is 30, while it is 20 in FOA and FA. The experiments are repeated 30 times.

Comparing with the FTSE100 index, we get the experiment results as shown in Tables 1–3 under *δ* = 0.05, 0.07, and 0.10, respectively, where the “Mean” column describes the average indicator value of optimal portfolios during repeated experiments, and the “Optimal” column describes the indicator value of optimal portfolios among repeated experiments. For the simplicity of data representation, the order of magnitude of EMR in the table is 10^−4^. The order of magnitude of DD is 10^−2^.

In Figures 3(a)–3(c), the histograms, respectively, show the specific asset structure of the optimal portfolio of 101 kinds of assets when the model upper bound = 0.05, 0.07, and 0.10 by the WOA algorithm. The histogram in Figure 3(d) shows the specific asset structure of FTSE100. From Figures 4–21, it can be seen that the line charts show the daily rate of return of the optimal portfolio of WOA, GWO, FOA, PSO, and FA when the model upper bound = 0.05, 0.07, and 1.10 and compared with the daily rate of return of FTSE100.

Figures 22–24 show the box plots of the net return, skewness, and Shannon's entropy obtained by five different algorithms when the model upper bound = 0.05, 0.07, and 0.10, respectively.

### 4.3. Numerical Analysis

We perform the numerical experiments of the multiconstraint portfolio optimization model under SSD by WOA, GWO, FOA, PSO, and FA. In this part, the performance of the portfolio obtained by each algorithm is evaluated through returns, diversity, risk, relative risk value, and other indicators.

First of all, it can be seen from Table 1 that the mean net return of portfolios obtained by WOA is 0.06737, while the portfolios obtained by GWO, FOA, PSO, and FA are 0.05907, 0.04590, 0.05327, and 0.05043, respectively, under *δ* = 0.05. Besides, the mean net return and EMR of portfolios obtained by WOA under different upper bounds are also higher than portfolios obtained by the other four algorithms. If only considering the optimization results, it is obvious that WOA has a better optimization capability than the other four algorithms under strong constraints. Although the mean SE of the portfolio obtained by WOA is slightly lower than the value obtained by FOA, the mean Sharpe ratio of FOA is much lower than the performance obtained by WOA. For investors, they tend to choose the portfolio with a higher Sharpe ratio when there is a little difference in SE respect. Meanwhile, the primary goal of investors is to obtain the maximum returns, while the returns of the portfolio obtained by FOA are far lower than that obtained by WOA. Taken together, FOA's results are unsatisfactory in that they give us a slight advantage in diversity at the expense of returns.

In Table 2, when *δ* = 0.07, the mean and optimal net returns of the portfolio obtained by WOA are higher than those obtained by the other four algorithms. In Table 3, the performance obtained by WOA is superior to that obtained by the other four algorithms under *δ* = 0.10. The numerical analysis results in Table 2 and Table 3 are similar to those in Table 1.

**Figure 3:**
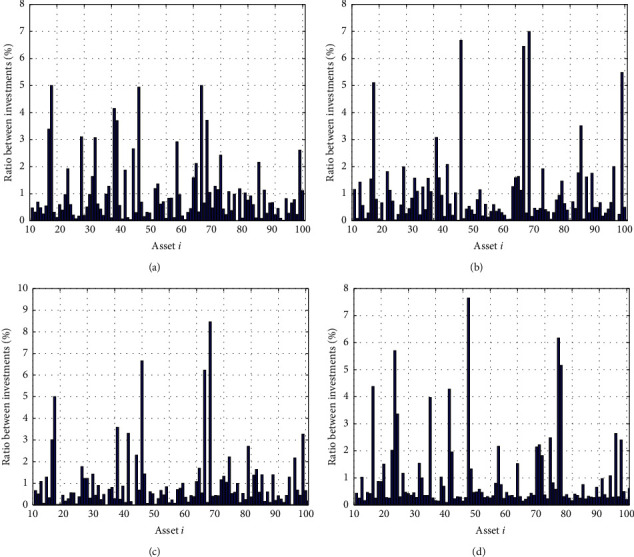
The specific asset structure of the optimal portfolio in (a) *δ* = 0.05, (b) *δ* = 0.07, and (c) *δ* = 0.10 for 101 assets by the WOA algorithm and the (d) FTSE100 index portfolio.

Secondly, it can be seen from Figures 3(a)–3(c) the 101 asset structure of the optimal portfolio obtained by the WOA algorithm under *δ* = 0.05, 0.07, and 0.10.  Figure 3(b) shows the asset portfolio obtained by FTSE100. The portfolio optimized by WOA has a more even distribution of assets, and its upper bound is lower than the upper bound of FTSE100, which proves that its diversity is better.

**Figure 4:**
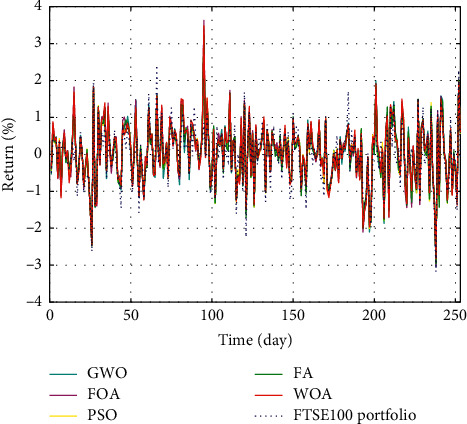
The returns of the optimal portfolio by WOA, GWO, FOA, PSO, and FA algorithms and the FTSE100 index portfolio in backtesting with *δ* = 0.05.

**Figure 5:**
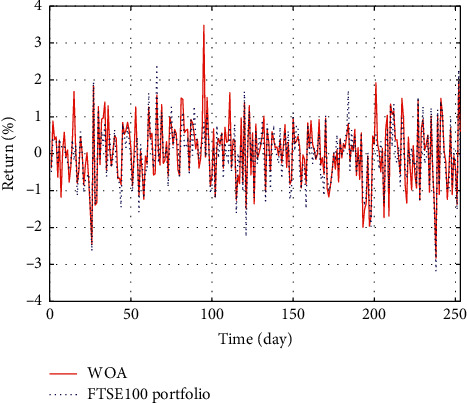
The returns of the optimal portfolio by the WOA algorithm and the FTSE100 index portfolio in backtesting with *δ* = 0.05.

**Figure 6:**
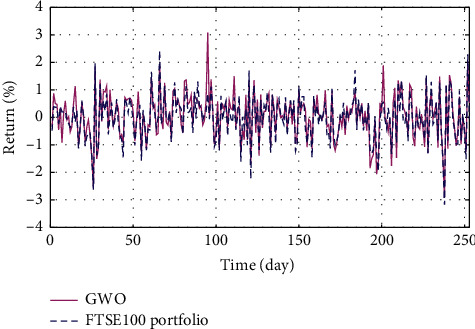
The returns of the optimal portfolio by the GWO algorithm and the FTSE100 index portfolio in backtesting with *δ* = 0.05.

**Figure 7:**
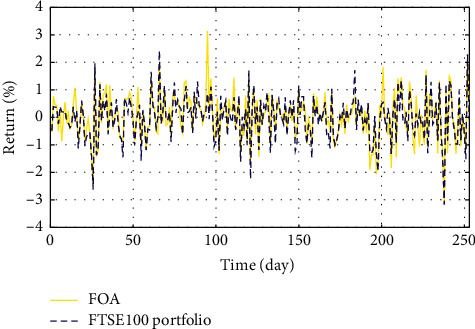
The returns of the optimal portfolio by the FOA algorithm and the FTSE100 index portfolio in backtesting with *δ* = 0.05.

**Figure 8:**
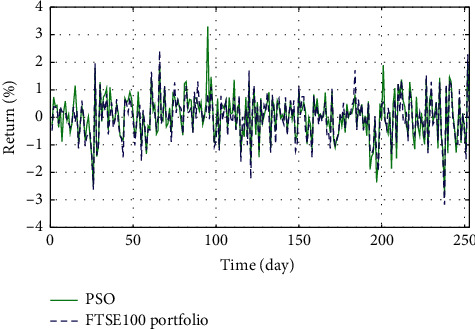
The returns of the optimal portfolio by the PSO algorithm and the FTSE100 index portfolio in backtesting with *δ* = 0.05.

**Figure 9:**
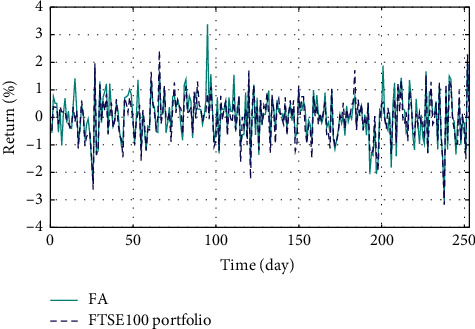
The returns of the optimal portfolio by the FA algorithm and the FTSE100 index portfolio in backtesting with *δ* = 0.05.

**Figure 10:**
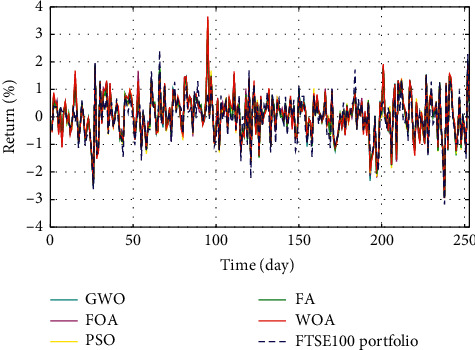
The returns of the optimal portfolio by WOA, GWO, FOA, PSO, and FA algorithms and the FTSE100 index portfolio in backtesting with *δ* = 0.07.

**Figure 11:**
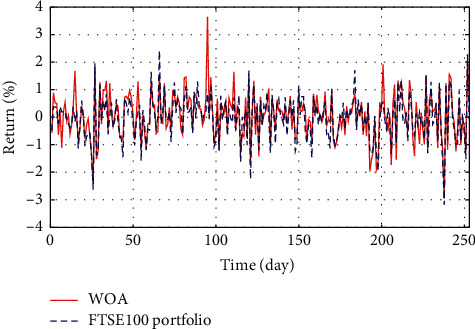
The returns of the optimal portfolio by the WOA algorithm and the FTSE100 index portfolio in backtesting with *δ* = 0.07.

**Figure 12:**
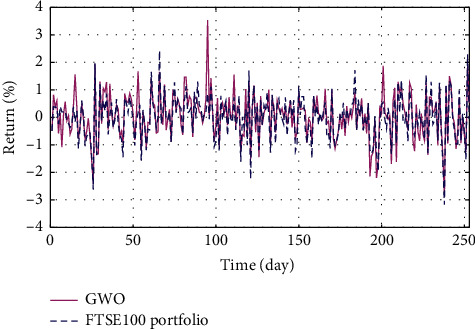
The returns of the optimal portfolio by the GWO algorithm and the FTSE100 index portfolio in backtesting with *δ* = 0.07.

**Figure 13:**
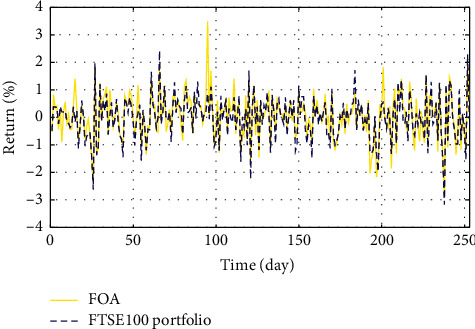
The returns of the optimal portfolio by the FOA algorithm and the FTSE100 index portfolio in backtesting with *δ* = 0.07.

**Figure 14:**
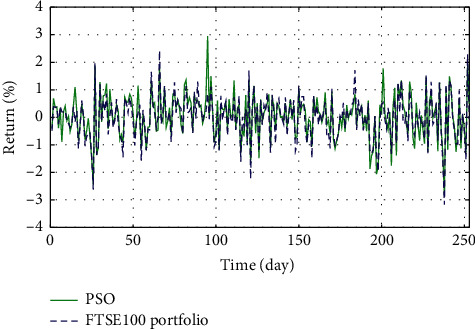
The returns of the optimal portfolio by the PSO algorithm and the FTSE100 index portfolio in backtesting with *δ* = 0.07.

**Figure 15:**
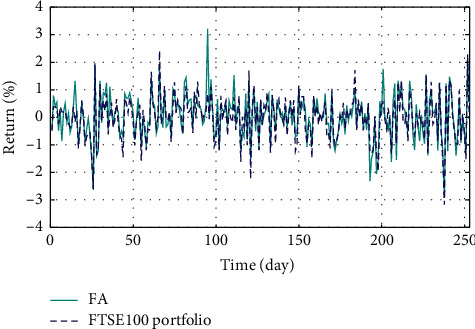
The returns of the optimal portfolio by the FA algorithm and the FTSE100 index portfolio in backtesting with *δ* = 0.07.

**Figure 16:**
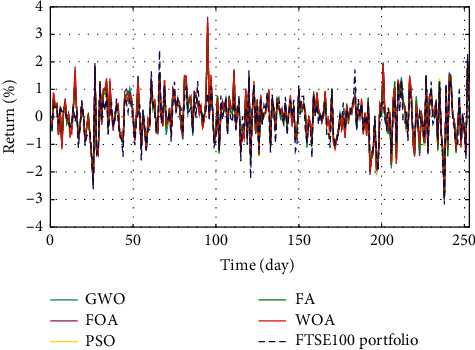
The returns of the optimal portfolio by WOA, GWO, FOA, PSO, and FA algorithms and the FTSE100 index portfolio in backtesting with *δ* = 0.10.

**Figure 17:**
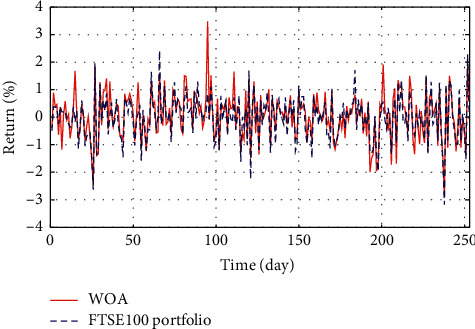
The returns of the optimal portfolio by the WOA algorithm and the FTSE100 index portfolio in backtesting with *δ* = 0.10.

**Figure 18:**
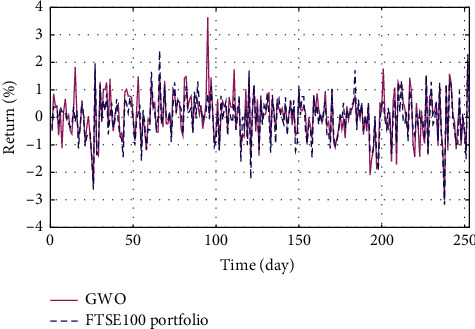
The returns of the optimal portfolio by the GWO algorithm and the FTSE100 index portfolio in backtesting with *δ* = 0.10.

**Figure 19:**
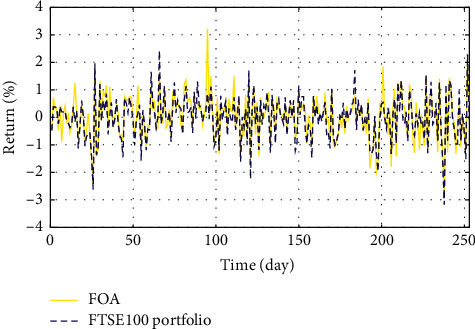
The returns of the optimal portfolio by the FOA algorithm and the FTSE100 index portfolio in backtesting with *δ* = 0.10.

**Figure 20:**
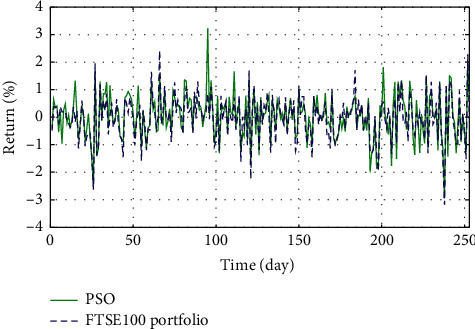
The returns of the optimal portfolio by the PSO algorithm and the FTSE100 index portfolio in backtesting with *δ* = 0.10.

**Figure 21:**
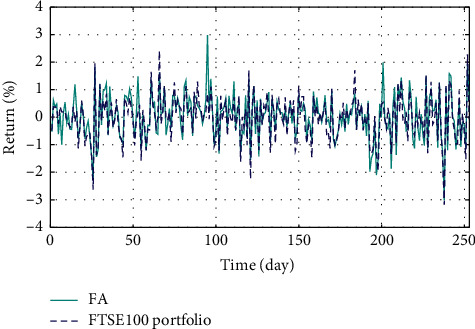
The returns of the optimal portfolio by the FA algorithm and the FTSE100 index portfolio in backtesting with *δ* = 0.10.

In Figures 4–21, the horizontal axis represents 253 trading days and the vertical axis represents the return rate. These figures show the daily rate of return of the optimal portfolio of WOA, GWO, FOA, PSO, and FA when *δ* = 0.05, 0.07, and 0.10 and compare it with the daily rate of return of FTSE100. It can be seen that the return rate of the portfolio optimized by these five different algorithms is mostly higher than the return rate of FTSE100 under different values of *δ*. From Figures 4–9, it is seen that when *δ* = 0.05, the return rate of the portfolio obtained by the five different algorithms is mostly better than the return rate of FTSE100 in 253 trading days. From Figures 10–15, it is seen that the portfolio return rate obtained by the five different algorithms is mostly better than the return rate of FTSE100 under *δ* = 0.07. From Figures 16–21, it is seen that the optimized portfolio return rate is mostly better than the return rate of FTSE100 under *δ* = 0.10. From Figures 4, 10, and 16, for approximately 80% of the trading days, the return rate line of the portfolio obtained by WOA (red line) is higher than the FTSE100's return rate line (blue dotted line). Compared with the return rate line of the portfolio obtained by other four kinds of algorithm, it can be proved that the returns of the portfolio optimized by WOA are higher than that of the other four algorithms.

**Figure 22:**
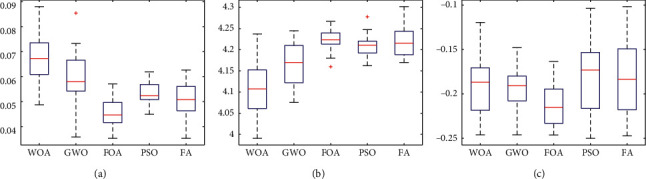
*δ* = 0.05. (a) Net Return. (b) Shannon's entropy. (c) Skewness.

**Figure 23:**
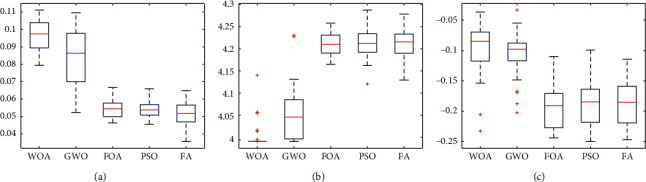
*δ* = 0.07. (a) Net Return. (b) Shannon's entropy. (c) Skewness.

**Figure 24:**
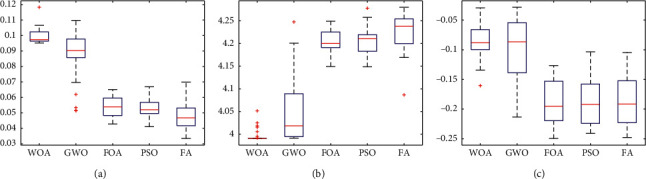
*δ* = 0.10. (a) Net Return. (b) Shannon's entropy. (c) Skewness.

Further, from Figures 22–24, we can see the box plots of net return, Shannon's entropy, and skewness of the portfolios optimized by the five different algorithms. It can be seen that when *δ* = 0.05, 0.07, and 0.10, respectively, the mean returns of the portfolio optimized by WOA are higher than that of the other four algorithms. For investors, they tend to choose the portfolio with a higher net return, so the portfolio optimized by WOA is a useful guideline to investors.

Although the mean Shannon's entropy of the portfolio obtained by WOA is slightly lower than the value obtained by the other four algorithms, the mean net return of WOA is the highest of these algorithms. It means that in order to achieve a higher net return, a part of the diversity needs to be sacrificed.

When *δ* = 0.05, the mean skewness of the portfolio optimized by WOA is only 0.0124 lower than the maximum of that obtained by the other four algorithms. When *δ* = 0.07 and 0.10, the portfolios optimized by WOA have a greater skewness than the other algorithms. At the same time, the mean net return of WOA is the highest of these algorithms. For investors, they prefer the portfolio with larger skewness and higher returns. Therefore, the portfolio obtained by WOA is more suitable for investors to choose.

In addition, besides using FTSE100 as the comparison criterion, equally weighted indicators are also used as the comparison object. When investors build portfolios, more reference schemes are provided. The indicators of FTSE100 and equal weight are also given in Table 4.

From Table 4, it can be seen that the net return under FTSE100 and equal weight is −0.1038 and −0.1057, which is lower than that optimized by these algorithms. Meantime, their skewness is all lower than that optimized by these algorithms. Their kurtosis is greater than 3, indicating that the solutions present a spike pulse. Their kurtosis is all higher than that optimized by these algorithms. By comparing with the performance indicators in the case of FTSE100 and equal weight, it indicates that the performance of the optimized portfolio by WOA has a greater reference value for investors.

The specific index weight data of the FTSE100 index are shown in Table 5. In this paper, we use the Sortino ratio, STARR ratio, Sharpe ratio, and IR to measure the relative risk value, which is used to quantify the net return or the excess mean return from taking on the unit risk. When the upper bound is set to 0.05, it can be seen from Table 1 that the mean Sortino ratio of portfolios obtained by WOA is 0.3004, while the portfolios obtained by GWO, FOA, PSO, and FA are 0.2930, 0.2732, 0.2817, and 0.2746, respectively. Besides, the mean STARR ratio, Sharpe ratio, and IR of portfolios obtained by WOA are also better than the other four algorithms, which means that the portfolio obtained by WOA has a better relative risk value. Above all, under several constraints, WOA has an efficient search ability to find the optimal portfolio that meets the constraints. In addition, in terms of the algorithm structure, WOA also has the advantages of a simple structure and few parameter settings. From the upper bound of results, the upper bound of WOA is higher than other algorithms, which indicates that WOA has a good convergence effect and strong local search ability.

Furthermore, the SSD optimization model proposed in this paper has a strong constraint on the solution. In other words, the existence of constraints will lead to infeasible regions in the search space of decision variables. In fact, in the field of finance, realistic constraints are pretty important. The existence of constraints will lead to infeasible regions in the search space of decision variables. The algorithm needs to balance constraints and optimization. Under strong constraints, the area of the feasible region is very small, and a large number of solutions are considered to be infeasible because they do not meet the constraints, which makes it a very difficult problem to produce feasible solutions. At the same time, the existence of constraints may also make the original problem produce many new local optima [48]. In this case, the optimization algorithm is easy to converge too quickly, resulting in the loss of population diversity in the early stage, and the global optimal solution cannot be found. Therefore, the strong constraint optimization problem has a high demand on the ability of the algorithm to jump out of the local optimum and the ability of global search [49]. According to the experimental results in this section, under different upper bounds, the value of the objective function obtained by WOA is better in both the optimal conditions [50, 51]. This shows that the performance of WOA is better than that of the other four algorithms in strongly constrained optimization problems, which has an excellent application prospect.

## 5. Conclusion

Since Fama and French [35] put forward the three-factor pricing model, there has been a boom in searching for factors in academic circles recently. Factor model is essentially an asset pricing model and can predict the future returns on assets to a certain extent. However, no matter how many factors there are, we should strive to achieve higher returns on the premise of minimizing portfolio risks, namely, asset allocation. With the development of machine learning, more and more advanced algorithms are used to explore the nonlinear relationship between factors and returns on assets. Similarly, machine learning can be applied to asset allocation.

In this paper, we propose a portfolio optimization model under SSD and several realistic constraints, and it is optimized by WOA. As a matter of fact, the real financial environment is much more complex. Therefore, how to broaden the practicability of the intelligent algorithm in a complex financial environment is still a long way to go. Based on FTSE100 index stock data, the experimental results prove the outstanding performance of WOA during harsh constraints.

However, the proposed portfolio optimization model only considers a single-period problem. In fact, portfolio construction is a continuous process. In the future work, establishing a multiperiod portfolio model and verifying the performance of WOA or other intelligent algorithms are feasible. Moreover, WOA has a comparatively huge rise space in global searching, and we find that the WOA has the problem of premature convergence. We will pay attention to this topic in the future research.

## Figures and Tables

**Algorithm 1:**
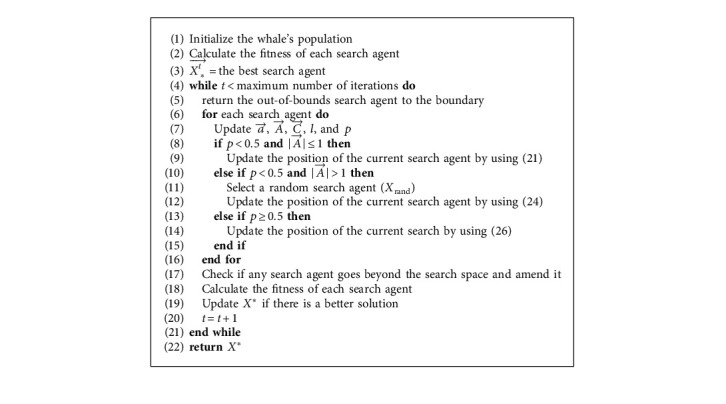
The main procedure of WOA.

**Algorithm 2:**
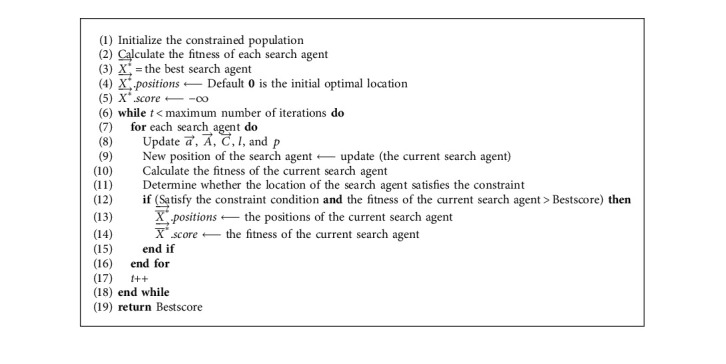
The pseudocode of WOA for solving the proposed model.

**Table 1:** The performance of the portfolio obtained by the various algorithms under *δ* = 0.05.

Indicators/algorithms	WOA		GWO		FOA		PSO		FA	
	Mean	Optimal	Mean	Optimal	Mean	Optimal	Mean	Optimal	Mean	Optimal
Net return	0.06737	0.08801	0.05907	0.08540	0.04590	0.05707	0.05327	0.06190	0.05043	0.06266
Skewness	−0.1925	−0.1958	−0.1940	−0.1787	−0.2118	−0.1719	−0.1819	−0.1533	−0.1801	−0.1017
Kurtosis	0.7343	0.8044	0.7090	0.6122	0.6726	0.8385	0.7681	0.9286	0.7851	0.8488
Shannon's entropy	4.1047	4.0839	4.1639	4.0754	4.2235	4.2332	4.2087	4.2044	4.2213	4.1845
Yager's entropy	−0.7950	−0.8218	−0.7589	−0.8384	−0.7075	−0.6827	−0.7142	−0.7639	−0.7040	−0.7360
EMR	6.9164	7.7089	6.5942	7.6058	6.0903	6.4979	6.3322	6.6825	6.2200	6.6440
VaR_*α*_	−0.01334	−0.01331	−0.01334	−0.01393	−0.01330	−0.01357	−0.01333	−0.01325	−0.01330	−0.01346
CVaR_*α*_	−0.01770	−0.01790	−0.01764	−0.01787	−0.01760	−0.01773	−0.01770	−0.01785	−0.01766	−0.01777
Downside deviation	0.2306	0.2316	0.2257	0.2040	0.2232	0.2384	0.2252	0.2269	0.2273	0.2512
Sortino ratio	0.3004	0.3327	0.2930	0.3728	0.2732	0.2725	0.2817	0.2945	0.2746	0.2644
STARR ratio	−0.03905	−0.04305	−0.03737	−0.04253	−0.03458	−0.03663	−0.03577	−0.03742	−0.03521	−0.03737
Sharpe ratio	3.2811	5.7412	2.3138	5.3432	0.7703	2.0340	1.5921	2.6265	1.3023	2.6582
Information ratio	0.1759	0.1921	0.1722	0.2060	0.1635	0.1617	0.1647	0.1695	0.1616	0.1536
Times	32.1304	33.7750	34.3511	34.2063	31.3813	31.3803	30.7534	32.6466	37.2835	36.3269
Upper bound	0.04997	0.05000	0.04931	0.04977	0.04689	0.04954	0.05335	0.05662	0.05260	0.06131

**Table 2:** The performance of the portfolio obtained by the various algorithms under *δ* = 0.07.

Indicators/algorithms	WOA		GWO		FOA		PSO		FA	
	Mean	Optimal	Mean	Optimal	Mean	Optimal	Mean	Optimal	Mean	Optimal
Net return	0.09664	0.1111	0.08346	0.1095	0.05426	0.06662	0.05378	0.06588	0.05123	0.06484
Skewness	−0.0962	−0.0389	−0.1058	−0.0841	−0.1906	−0.1097	−0.1858	−0.2220	−0.1854	−0.1634
Kurtosis	0.9434	0.9932	0.9227	0.9654	0.7533	0.9873	0.7582	0.6570	0.7525	0.9273
Shannon's entropy	4.0016	3.9905	4.0554	3.9967	4.2113	4.2082	4.2105	4.1913	4.2097	4.1701
Yager's entropy	−0.8672	−0.8648	−0.8357	−0.8910	−0.7124	−0.7182	−0.7105	−0.7131	−0.7156	−0.7534
EMR	8.0035	8.5137	7.5014	8.4974	6.3716	6.8292	6.3506	6.8817	6.2507	6.7399
VaR_*α*_	−0.01333	−0.01361	−0.01329	−0.01343	−0.01330	−0.01301	−0.01248	−0.01247	−0.01243	−0.01214
CVaR_*α*_	−0.01780	−0.01782	−0.01769	−0.01791	−0.01763	−0.01782	−0.01636	−0.01623	−0.01634	−0.01641
Downside deviation	0.2414	0.2496	0.2434	0.2390	0.2267	0.2268	0.2263	0.2090	0.2247	0.2305
Sortino ratio	0.3317	0.3410	0.3091	0.3554	0.2814	0.3012	0.2812	0.3291	0.2787	0.2922
STARR ratio	−0.04496	−0.04776	−0.04238	−0.04742	−0.03613	−0.03833	−0.03881	−0.04238	−0.03823	−0.04106
Sharpe ratio	6.6455	8.2492	5.1370	8.1545	1.7145	3.1584	1.6535	3.1410	1.3999	2.9601
Information ratio	0.1860	0.1897	0.1762	0.1955	0.1654	0.1693	0.1647	0.1891	0.1637	0.1693
Times	36.1142	37.3528	37.9714	38.7565	34.0302	35.6235	30.8553	30.7140	37.0023	39.2392
Upper bound	0.06627	0.07000	0.06516	0.07000	0.05363	0.06101	0.05348	0.04946	0.05294	0.05344

**Table 3:** The performance of the portfolio obtained by the various algorithms under *δ* = 0.10.

Indicators/algorithms	WOA		GWO		FOA		PSO		FA	
	Mean	Optimal	Mean	Optimal	Mean	Optimal	Mean	Optimal	Mean	Optimal
Net return	0.1001	0.1182	0.08775	0.1097	0.05386	0.06503	0.05337	0.06692	0.04820	0.06993
Skewness	−0.08742	−0.07992	−0.1029	−0.05443	−0.1898	−0.1845	−0.1880	−0.1577	−0.1860	−0.1732
Kurtosis	0.9275	0.7266	0.8847	0.9680	0.7697	0.8485	0.7766	0.8309	0.7746	0.6532
Shannon's entropy	3.9961	3.9906	4.0519	3.9914	4.2038	4.1722	4.2069	4.1627	4.2230	4.0865
Yager's entropy	−0.8616	−0.8613	−0.8237	−0.8966	−0.7190	−0.7411	−0.7146	−0.7499	−0.7034	−0.8342
EMR	8.0891	8.8274	7.6398	8.4398	6.3660	6.7649	6.3501	6.8179	6.1376	6.9920
VaR_*α*_	−0.01349	−0.01368	−0.01350	−0.01398	−0.01337	−0.01329	−0.01089	−0.01110	−0.01088	−0.01086
CVaR_*α*_	−0.01780	−0.01769	−0.01779	−0.01783	−0.01772	−0.01751	−0.01504	−0.01512	−0.01498	−0.01481
Downside deviation	0.2546	0.2480	0. 2471	0.2655	0.2327	0.2414	0.2262	0.2513	0.2251	0.2403
Sortino ratio	0.3190	0.3558	0.3103	0.3177	0.2746	0.2802	0.2811	0.2712	0.2732	0.2909
STARR ratio	−0.04543	−0.04989	−0.04295	−0.04731	−0.03593	−0.03863	−0.04220	−0.04507	−0.04095	−0.04718
Sharpe ratio	7.0194	9.0582	5.5788	8.0460	1.6596	3.0298	1.6079	3.2149	1.0754	3.5891
Information ratio	0.1808	0.1978	0.1767	0.1799	0.1624	0.1650	0.1650	0.1620	0.1613	0.1693
Times	34.1131	35.3522	34.3511	34.2063	33.9993	35.9620	31.3336	33.5159	38.0149	39.8047
Upper bound	0.07952	0.08456	0.07515	0.08125	0.05307	0.05465	0.05357	0.05872	0.05226	0.06554

**Table 4:** The indicators of FTSE100 and equal weight.

	Net return	VaR_*α*_	CVaR_*α*_	Skewness	Kurtosis	SE
FTSE100	−0.1038	−0.0134	−0.0179	−0.2501	3.9934	3.9905
Equal weight	−0.1057	−0.0142	−0.0178	−0.3211	3.5008	4.6150

**Table 5:** The specific index weight data of the FTSE100 index.

Constitution	Index weight (%)
3i Group	0.43
Anglo American	1.02
Ashtead Group	0.46
AstraZeneca	4.38
Aviva	0.87
Barclays	1.51
Berkeley Group Holdings	0.25
BP	5.70
British Land Co.	0.30
Bunzl	0.47
Carnival	0.32
Coca-Cola HBC AG	0.28
CRH	1.00
DCC	0.35
Direct Line Insurance Group	0.26
Evraz	0.14
Ferguson	0.69
GlaxoSmithKline	4.28
GVC Holdings	0.23
Hargreaves Lansdown	0.29
Hiscox	0.27
Imperial Brands	1.33
InterContinental Hotels Group	0.48
Intertek Group	0.46
Johnson Matthey	0.31
Land Securities Group	0.34
Lloyds Banking Group	2.17
Marks and Spencer Group	0.24
Micro Focus International	0.34
Morrison (WM) Supermarkets	0.27
Next	0.31
Ocado Group	0.21
Pearson	0.43
Prudential	2.14
RELX	1.82
Rightmove	0.23
Rolls-Royce Holdings	0.82
Royal Dutch Shell A	6.17
RSA Insurance Group	0.31
Sainsbury (J)	0.25
Scottish Mortgage Inv Tst	0.40
Severn Trent	0.25
Smith (DS)	0.22
Smurfit Kappa Group	0.29
SSE	0.65
Standard Chartered	0.97
Taylor Wimpey	0.26
TUI AG	0.29
United Utilities Group	2.30
Whitbread	0.49
WPP	0.61
Admiral Group	0.25
Antofagasta	0.16
Associated British Foods	0.42
Auto Trader Group	0.25
BAE Systems	0.86
Barratt Developments	0.27
BHP Group Plc	2.02
British American Tobacco	3.36
BT Group	1.17
Burberry Group	0.42
Centrica	0.45
Compass Group	1.54
Croda International	0.35
Diageo	3.97
Easyjet	0.16
Experian	1.03
Fresnillo	0.09
Glencore	1.96
Halma	0.30
Hikma Pharmaceuticals	0.14
HSBC Hldgs	7.65
Informa	0.46
International Consolidated Airlines Group	0.58
ITV	0.27
Kingfisher	0.26
Legal & General Group	0.80
London Stock Exchange Group	0.75
Melrose Industries	0.46
Mondi	0.35
National Grid	1.53
NMC Health	0.14
Paddy Power Betfair	0.30
Persimmon	0.36
Reckitt Benckiser Group	2.22
Rentokil Initial	0.37
Rio Tinto	2.48
Royal Bank Of Scotland Group	0.58
Royal Dutch Shell B	5.16
Sage Group	0.38
Schroders	0.16
Segro	0.35
Smith & Nephew	0.75
Smiths Group	0.32
Spirax-Sarco Engineering	0.27
St. James's Place	0.29
Standard Life Aberdeen	0.38
Tesco	1.08
Unilever	2.64
Vodafone Group	2.40
Wood Group (John)	0.20

## Data Availability

The composition of FESE100 is obtained from the London stock exchange (http://www.londonstockexchange.com). The daily closing price of the constituent shares and dividends is obtained from Google Finance (http://www.google.com/finance).

## Abbreviations

WOA: Whale optimization algorithm
GWO: Gray wolf optimizer
FOA: Fruit fly optimization algorithm
PSO: Particle swarm optimization
FA: Firefly algorithm
EMR: Excess mean return
DD: Downside deviation
STARR: STARR ratio
IR: Information ratio
SE: Shannon's entropy
YE: Yager's entropy
VaR: Value at risk
CVaR: Conditional value at risk
SD: Stochastic dominance
FSD: First-order stochastic dominance
SSD: Second-order stochastic dominance
ASD: Almost stochastic dominance
M-V: Mean-variance.
